# Protective effects of icariin in atherosclerotic cardiovascular disease: mechanistic basis, post-ischemic protection and translational limitations

**DOI:** 10.1080/13880209.2026.2740872

**Published:** 2026-10-10

**Authors:** Xuefei Wang, Zongliang Yu, Xiaoya Li, Lanqing Hu, Changxin Sun, Yajie Wang, Liang Li, Min Wu, Longtao Liu

**Affiliations:** ^a^ Beijing University of Chinese Medicine, Beijing, China; ^b^ Department of Cardiology, Guang’anmen Hospital, China Academy of Chinese Medical Sciences, Beijing, China; ^c^ State Key Laboratory of Traditional Chinese Medicine Syndrome/National Clinical Research Center for Chinese Medicine Cardiology, Xiyuan Hospital, China Academy of Chinese Medical Sciences, Beijing, China; ^d^ Department of Geriatrics, Zhongshan Hospital of Traditional Chinese Medicine, Zhongshan, China

**Keywords:** Icariin, Epimedii Folium, atherosclerotic cardiovascular disease, pharmacokinetics, vascular injury

## Abstract

**Context:**

Icariin (ICA), a prenylated flavonol glycoside from Epimedii Folium, has been investigated in experimental models relevant to atherosclerosis, myocardial infarction, and ischemic tissue injury.

**Objectives:**

This review evaluates experimental evidence on ICA in atherosclerotic plaque biology, post-myocardial infarction remodeling, myocardial ischemia-reperfusion injury, and cerebral ischemic injury.

**Methods:**

PubMed and the Web of Science Core Collection were searched from database inception to 19 August 2026, and studies were selected using predefined eligibility criteria. Targeted searches also covered pharmacokinetics, metabolism, toxicology, botanical source, quality control, formulation, and delivery.

**Results:**

In atherosclerosis models, ICA reduced endothelial inflammatory activation, macrophage lipid uptake, vascular smooth muscle cell (VSMC) proliferation and migration, and lesion burden. Several mechanistic links were tested using pharmacological or genetic perturbation. *In vivo* studies primarily characterized lesion burden and circulating lipids, with less direct assessment of plaque composition and stability. Post-myocardial infarction and ischemic injury studies reported reductions in fibrosis and ischemic tissue injury, together with reduced mitochondrial injury and changes in inflammatory signaling, apoptosis, and ferroptosis-related processes. Functional improvement was also reported in several models. Few studies measured parent ICA or metabolite concentrations in plaque, myocardium, or brain in the same experiments in which pathological outcomes were assessed.

**Conclusions:**

Future studies should quantify parent ICA and active metabolites in diseased tissues while assessing plaque composition and stability in the same experiments, so that vascular effects can be related to measured *in vivo* exposure.

## Introduction

Cardiovascular disease remains a leading cause of death and disability worldwide. Although age-standardized cardiovascular mortality and morbidity have declined over recent decades, population aging and persistent exposure to cardiometabolic risk factors have increased the absolute number of affected individuals (Diseases and Injuries 2020; Roth et al. 2020). Atherosclerotic diseases, particularly ischemic heart disease and stroke, account for a large proportion of cardiovascular mortality. Ischemic heart disease contributes approximately half of cardiovascular deaths, and stroke accounts for about one-quarter, with ischemic mechanisms responsible for a substantial share of stroke cases (Roth et al. 2020; Nedkoff et al. 2023). Atherosclerosis develops through lipid retention, endothelial injury, immune and inflammatory responses, and vascular-wall remodeling (Libby 2021). Current prevention and treatment strategies include lifestyle modification, blood-pressure control, lipid-lowering therapy and antithrombotic treatment (Visseren et al. 2021), yet substantial cardiovascular risk persists. Experimental studies have reported effects of ICA on lipid metabolism, vascular inflammation, and ischemic tissue injury in models of atherosclerosis, post-MI remodeling, and ischemia–reperfusion. These experimental settings differ in pathological stage, treatment timing, and the drug exposures achieved *in vivo*.

Epimedii Folium is the dried leaf medicinal material obtained from *Epimedium brevicornu* Maxim., *Epimedium sagittatum* (Siebold & Zucc.) Maxim., *Epimedium pubescens* Maxim., *Epimedium koreanum* Nakai and *Epimedium wushanense* T.S. Ying (Berberidaceae). It has long been used in traditional Chinese medicine to tonify kidney yang, strengthen bones and tendons, and dispel wind-dampness. Modern studies have identified multiple prenylated flavonoids in *Epimedium* species, among which icariin is one of the most extensively investigated constituents (Fang and Zhang 2017; Wang et al. 2021; Bi et al. 2022). Icariin has the molecular formula C33H40O15 and a molecular weight of approximately 676.7 g/mol and is classified as a prenylated flavonol glycoside (He et al. 2020). Botanical origin, geographic source, harvesting and processing influence the major flavonoid profile of medicinal *Epimedium* materials (Xue et al. 2023). Icariin has been examined in experimental myocardial ischemia-reperfusion, myocardial remodeling, endothelial injury, atherosclerosis and ischemic brain injury (Shi et al. 2018; Liu S et al. 2021; Huang et al. 2023; Jia et al. 2023; Wang X et al. 2023; Zhong et al. 2024; Zhou et al. 2024). Previous reviews have addressed the cardiovascular actions of ICA and its metabolites, its biomedical and tissue-engineering applications, and more recent multi-disease pharmacology (Zeng et al. 2022; Seyedi et al. 2023; Li S et al. 2026). The present review focuses specifically on ASCVD-related evidence and separates plaque-stage atherosclerosis, post-MI remodeling, myocardial I/R injury, and cerebral ischemic injury. It further evaluates these studies in relation to experimental perturbation, parent-compound and metabolite exposure, safety, and formulation.

## Materials and methods

### Review design and literature search

For this narrative review, PubMed and the Web of Science Core Collection were systematically searched from database inception to 19 August 2026, and studies were selected according to predefined eligibility criteria. The core search combined icariin with controlled vocabulary and free-text terms for atherosclerosis, coronary and myocardial ischemic disease, cerebral ischemia, peripheral arterial disease, endothelial dysfunction, vascular calcification, foam-cell formation and oxidized low-density lipoprotein injury. Targeted searches addressed pharmacokinetics, metabolism, toxicology, safety, formulation, delivery, botanical source and quality control. No language or study-design limits were applied at the database-search stage. Reference lists of eligible original studies and relevant reviews were screened manually. Supplementary full-text and reference-list cross-checking identified six additional eligible experimental studies, which were incorporated into the narrative synthesis. Authoritative pharmacopeial sources were also consulted for botanical nomenclature and quality-control standards. Search records were imported into EndNote and deduplicated before screening. A PRISMA 2020 checklist is provided in Supplementary Material 1, complete database strategies are provided in Supplementary Material 2, and the study-selection process is shown in Figure 1. Supplementary Material 1 reports the PRISMA 2020 items applicable to the search and study-selection process and marks items outside the scope of this narrative review as not applicable.

**Figure 1.**
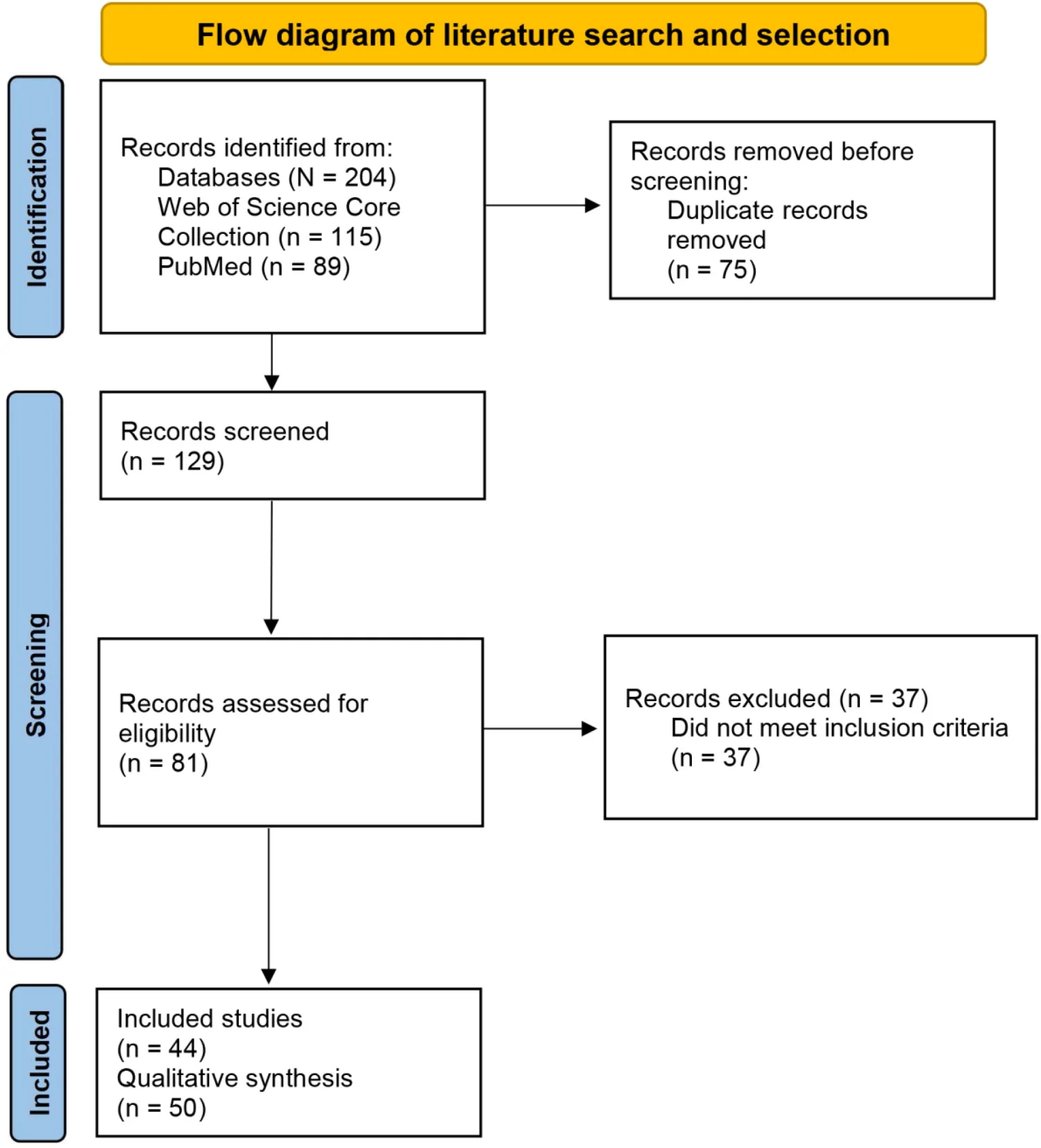
Flow diagram of database searching and study selection. PubMed and the Web of Science Core Collection were searched from database inception through 19 August 2026.

### Eligibility criteria

#### Inclusion criteria

Studies were included when they met at least one of the following conditions: (1) original *in vivo* or *in vitro* studies administering parent icariin in models of atherosclerosis, myocardial infarction, myocardial or cerebral ischemia-reperfusion injury, ischemic stroke or ASCVD-relevant vascular-cell injury. (2) original pharmacokinetic, metabolic, toxicological, formulation or delivery studies that informed icariin exposure or translational feasibility. (3) studies directly administering icariin-related metabolites, provided that the compound tested was clearly identified and interpreted separately from parent icariin. Selected studies without icariin administration were used only when they provided mechanistic context necessary to interpret pathways discussed in the review and were not treated as evidence of icariin efficacy.

#### Exclusion criteria

Studies were excluded when they were unrelated to the scope of the review, reported chemical or material characterization without pharmacological or translational relevance, or lacked sufficient methodological information for interpretation. Multicomponent *Epimedium* preparations were excluded from the core ICA efficacy synthesis when ICA-specific effects could not be isolated; selected extract studies were retained only as contextual evidence for source-related safety or quality-control issues. Duplicate publications, conference abstracts and editorials were excluded from the primary experimental synthesis.

### Study selection

Two reviewers independently screened titles and abstracts and assessed potentially eligible full-text articles. Disagreements were resolved through discussion, with a third reviewer consulted when necessary. Information on study design, experimental models, ICA intervention, main outcomes and mechanistic experiments was extracted from the included studies and checked by a second reviewer. When interpreting the evidence, we considered model relevance, adequacy of controls, the reporting of dose and exposure, and whether proposed mechanisms were tested using pharmacological or genetic approaches. Cell-culture findings were distinguished from *in vivo* lesion data, while myocardial and cerebral ischemic injury studies were interpreted as evidence of post-ischemic tissue injury and repair. No formal study-level risk-of-bias score was assigned because this was a narrative synthesis; methodological strengths and limitations were assessed qualitatively using the criteria above. Supplementary Material 3 provides a SANRA assessment of this review, whereas the included studies were appraised qualitatively using the methodological criteria described above.

## Botanical source, phytochemistry and quality control

### Botanical sources and traditional use

Epimedii Folium has a long history of medicinal use in traditional Chinese medicine. It has traditionally been used to tonify kidney yang, strengthen bones and tendons, and dispel wind-dampness. Its medicinal use can be traced to the Shennong Bencao Jing (The Divine Farmer’s Materia Medica), and the herb has also been used in other East Asian medical systems, including those of Korea and Japan (Wang et al. 2021; Bi et al. 2022). In modern pharmacopeial and herbal nomenclature, *Epimedium*-type medicinal materials mainly refer to the dried leaves of plants in the genus *Epimedium*, commonly denoted as Epimedii Folium or Herba Epimedii (Fang and Zhang 2017; Xue et al. 2023). *Epimedium* species are widely distributed in Asia, with China being an important center of distribution. Species, origin and processing methods can markedly affect the composition and content of flavonoids (Xue et al. 2023). For consistency with pharmacopeial nomenclature, the medicinal leaf material is referred to as Epimedii Folium throughout this review, and the isolated compound as icariin or ICA.

### Phytochemical composition

Plants of the genus *Epimedium* are mostly perennial herbs, and their dried leaves are the main medicinal part. The chemical profile is dominated by flavonoids, especially prenylated flavonoid glycosides. Studies of *Epimedium* chemistry have shown that species, medicinal part and processing method can all alter the flavonoid composition of *Epimedium*-derived materials (Wang K et al. 2023; Xue et al. 2023; Zhang et al. 2023). The distribution of constituents also differs among leaves, stems and rhizomes. Leaves usually contain higher levels of flavonoid glycosides, while stems and rhizomes may contain related derivatives or flavonoids with different skeletons (Zhou et al. 2012; Zhou et al. 2021; Wang K et al. 2023). This heterogeneity affects content determination, pharmacological interpretation and quality evaluation, making it necessary for icariin studies to specify the plant source, medicinal part and extraction procedure.

### Icariin and its metabolites

Icariin is one of the most extensively studied prenylated flavonol glycosides in *Epimedium*. Its structure contains a glucose moiety at C-3, a rhamnose moiety at C-7, a methoxy group at C-4 and a prenyl group at C-8 (He et al. 2020). *In vivo* and *in vitro* metabolism can generate related compounds, including icariside I, icariside II and icaritin, which may differ from the parent compound in absorption, tissue distribution and pharmacological activity (Wu et al. 2016; Szabó et al. 2022). Studies in which an icariin-related compound was administered directly are identified by compound and discussed separately from experiments using parent ICA. The chemical structures of icariin and representative related flavonoids are shown in Figure 2.

**Figure 2.**
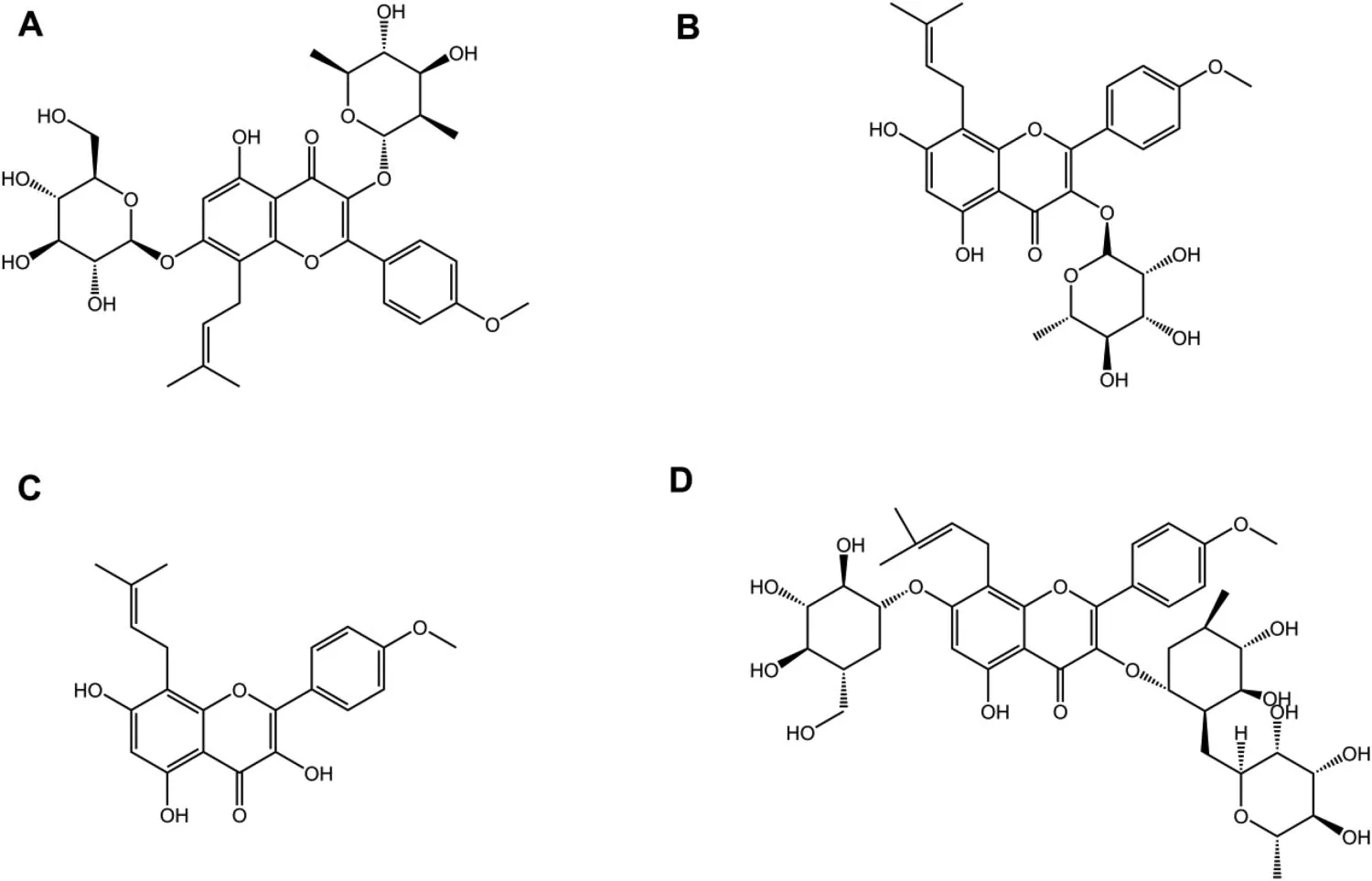
Chemical structures of representative prenylated flavonoids discussed in this review. A, icariin; B, baohuoside I (icariside II); C, icaritin; D, epimedin C. The authors redrew all four structures using ChemDraw with consistent bond, label, and stereochemistry settings.

### Preparation and quality control

The industrial preparation of icariin generally relies on cultivated or collected *Epimedium* plant materials. Hot-water and alcohol extraction remain common concentration methods, whereas ultrasound-assisted, microwave-assisted and ultra-high-pressure extraction, as well as biotransformation, have been used to improve extraction efficiency, conversion efficiency or the preparation of specific flavonoid metabolites (Zhang et al. 2023; Zhu et al. 2024). Studies of biotransformation and biosynthesis have described sequential conversion relationships among *Epimedium* flavonoids, including icariin-related glycosides and aglycones, which are relevant to the accessibility of active constituents and their pharmacokinetic behavior (Szabó et al. 2022; Zhu et al. 2024).

Quality control of *Epimedium* materials is complicated by heterogeneous botanical sources and variable flavonoid contents. Evaluation commonly includes icariin together with epimedin A, epimedin B and epimedin C, and substantial variation has been reported across species, geographical sources and commercial samples (Xue et al. 2023). The 2025 Pharmacopeia of the People’s Republic of China provides the current national standard for Epimedii Folium (Chinese Pharmacopoeia 2025). Studies using purified ICA should report the supplier, lot information, purity and analytical verification when available. Studies using *Epimedium* extracts or enriched fractions also need botanical authentication, extraction procedures, chemical fingerprints, major constituents and batch consistency.

## Mechanistic basis of icariin in atherosclerotic plaque biology

Retention of apoB-containing lipoproteins in the arterial intima is an early event in atherogenesis and promotes sustained local inflammation. Endothelial activation facilitates monocyte recruitment. Within the developing lesion, macrophages accumulate lipid and VSMCs undergo phenotypic changes. ICA studies have examined these cellular processes, with additional work addressing lipid metabolism and RNA-mediated regulation. The relevant evidence is summarized in Table 1.

**Table 1.** Preclinical studies of icariin and selected metabolites in ASCVD-related vascular, myocardial infarction, cardiac ischemia–reperfusion and cerebral ischemia models.

ASCVD-related setting	Experimental model	Compound, dose and timing	Main findings	Ref.
Vascular, endothelial and plaque models	Ox-LDL-treated HUVECs	ICA 10–40 μM	ICAM-1, VCAM-1, E-selectin and THP-1 adhesion↓.	(Hu et al. 2015)
	ApoE⁻/⁻ mice and LPC-treated HUVECs	*In vivo*: ICA 10 or 30 mg/kg/day. *In vitro*: ICA 1–10 μM	PRMT I and ADMA↓; DDAH II activity and vasorelaxation↑.	(Xiao et al. 2015)
	ApoE⁻/⁻ mice and LPC-treated HUVECs	*In vivo*: ICA 10 or 30 mg/kg. *In vitro*: ICA 1–10 μM	Aortic ROS and lesion area↓; eNOS and NO↑.	(Xiao et al. 2017)
	Homocysteine-treated HUVECs and rats	*In vivo*: ICA 20 mg/kg/day orally for 42 days. *In vitro*: ICA 0.1–5 μM.	ROS and endothelial senescence↓; Akt/eNOS signaling and NO↑.	(Xiao-Hong et al. 2013)
	H₂O₂-treated HUVECs with DTT to intensify ER stress	ICA 100 μM	Viability, adhesion, SOD and GSH-Px↑; apoptosis and ER-stress markers↓.	(Wang et al. 2019)
	GSNO-treated EA.hy926 endothelial cells	ICA 0.1, 1 or 10 μM for 48 h. LY294002 was used for pathway testing	Cell survival and Akt/eNOS↑; ROS, p53 and caspase-3↓.	(Huang et al. 2016)
	Rat bone-marrow-derived endothelial progenitor cells exposed to 25 mM glucose	ICA 0.01–1 μM. Maximal functional response at 1 μM	Proliferation, migration, tube formation and Akt/eNOS/NO↑.	(Chen S et al. 2019)
	Streptozotocin-induced type 1 diabetic rats, isolated aortas and endothelial cells	*In vivo*: ICA 20, 40 or 80 mg/kg/day. *Ex vivo* and *in vitro*: ICA 3, 10 or 30 μM	PPARα/SIRT1/AMPK/eNOS signaling and vasorelaxation↑; ER stress↓.	(Yao et al. 2021)
	Streptozotocin-induced type 1 diabetic rats and high-glucose-treated HUVECs	*In vivo*: ICA 20, 40 or 80 mg/kg/day. *In vitro*: ICA 3, 10 or 30 μM	GPER/SIRT1↑; HMGB1 and RAGE/TLR4/NF-κB signaling↓.	(Yao et al. 2024)
	Ox-LDL-treated HUVECs	ICA 10 μM	EndMT and migration↓; H19/miR-148b-3p/ELF5 signaling altered.	(Liu S et al. 2021)
	High-fat-diet-fed ApoE⁻/⁻ mice and ox-LDL-treated HUVECs	*In vivo*: ICA 40 or 80 mg/kg/day. *In vitro*: ICA 18.5–73.9 μM	Lesion burden, Fe²⁺ and lipid peroxidation↓; TFEB/autophagy-related activity and GPX4/FTH1↑.	(Wang X et al. 2023)
	Ox-LDL-treated HUVECs	ICA 10–40 μM	Bcl-2↑; caspase-3 and endothelial apoptosis↓.	(Hu et al. 2018)
	High-cholesterol-diet-fed ApoE⁻/⁻ mice and LPS-stimulated RAW264.7 cells	*In vivo*: ICA 30 or 60 mg/kg/day. *In vitro*: ICA 0.005 or 0.01 μM	Lesion macrophages and CX3CL1/CX3CR1-dependent chemotaxis↓.	(Wang et al. 2016)
	LPS- and ox-LDL-treated THP-1 macrophages	ICA 0.8–20 μM	Lipid uptake, CD36 and foam-cell formation↓; SR-BI↑.	(Yang et al. 2015)
	High-cholesterol diet plus vitamin D₃-induced atherosclerotic rats	ICA 30 or 60 mg/kg/day	Atherogenic lipids, inflammatory markers and p38 MAPK↓; HDL-C and SOD↑.	(Hu Y et al. 2016)
	High-fat-diet-fed ApoE⁻/⁻ mice and ox-LDL-treated human aortic VSMCs	*In vivo*: ICA 40 mg/kg. *In vitro*: ICA 10 μM	Plaque formation, VSMC proliferation and migration↓; apoptosis↑.	(Huang et al. 2023)
	Ox-LDL-treated VSMCs	ICA 10–40 μM	Proliferation, G1/S progression, PCNA and ERK1/2 phosphorylation↓.	(Hu Y et al. 2016)
	High-fat-diet-fed ApoE⁻/⁻ mice and ox-LDL-treated human aortic VSMCs	*In vivo*: ICA 20, 40 or 80 mg/kg. *In vitro*: ICA 10 μM	PKCβI activation, cyclin D1, MMP-9, proliferation and migration↓.	(Zhang et al. 2021)
	Ox-LDL-treated human aortic VSMCs	ICA 10 μM	H19–HuR signaling and cyclin D1/MMP-9 mRNA stability↓; proliferation and migration↓.	(Zhang et al. 2025)
	Homocysteine-treated VSMCs	ICA 0.30 or 0.59 μM (0.2 or 0.4 mg/L) for 48 h	Early apoptosis and GRP78 mRNA↑.	(Shen and He 2009)
	Cholesterol-fed rabbits with atherosclerosis	ICT 10 mg/kg/day orally for 2 months	Macrophage accumulation and MMP-1↓; collagen type I↑.	(Zhang et al. 2016)
	β-Glycerophosphate-induced mouse VSMCs	ICA 100–10,000 μM (0.1–10 mmol/L)	ALP, Runx2 and mineralization↓.	(He et al. 2023)
	Rat thoracic-aortic VSMCs exposed to high glucose and H₂O₂	ICA was started 1 week after calcification induction. The concentration was not available in the indexed abstract	ALP, calcium deposition and osteogenic markers↓; α-SMA↑.	(Bai et al. 2021)
	High-fat-diet-fed ApoE⁻/⁻ mice in two profiling studies	ICA 40 mg/kg/day for 12 weeks	Aortic lesion area↓; miRNA, lncRNA and mRNA profiles altered.	(Zhang et al. 2018, 2019)
	Cholesterol-fed rabbits	ICA 5 mg/kg	Plaque burden, platelet adhesion/aggregation and PAI-1/tPA imbalance↓.	(Zhang et al. 2013)
Myocardial infarction and cardiac ischemia–reperfusion models	Rat *in vivo* myocardial I/R	ICA postconditioning. Dose and exact timing were not reported in the indexed abstract	Infarct size and apoptosis↓; PI3K/Akt activation↑.	(Meng et al. 2015)
	Isolated Wistar rat hearts with 30 min global ischemia and 60 min reperfusion	ICA 5 or 10 μM	Cardiac function and SOD↑; infarct size, CK-MB, LDH, MDA and apoptosis↓.	(Ke et al. 2015)
	Streptozotocin-induced diabetic rats with 30 min left anterior descending coronary artery occlusion and 120 min reperfusion	ICA-treated diabetic I/R group. Dose requires confirmation from the Chinese full text	NO, SOD, GSH-Px and Bcl-2↑; myocardial injury and apoptosis markers↓.	(Hu et al. 2015)
	Primary neonatal rat cardiomyocytes	ICA 1 or 10 μM for 24 h before hypoxia/reoxygenation	Apoptosis and NF-κB signaling↓; Bcl-2↑.	(Xiang et al. 2015)
	Permanent coronary artery ligation in rats	ICA 3, 6, 12 or 20 mg/kg/day. The main mechanistic analyses used 12 mg/kg/day intraperitoneally for 28 days	Ventricular remodeling, apoptosis, collagen I/III, CD147 and MMP-9↓; LVEF↑.	(Shi et al. 2018)
	Myocardial infarction mice	ICA 60 mg/kg orally once daily for 28 days	CD8⁺/Th1 cells, inflammation, fibrosis and apoptosis↓; Treg and Nrf2/HO-1↑.	(Sai et al. 2022)
	left anterior descending coronary artery-ligation rats and IL-4-treated RAW264.7 macrophages	*In vivo*: ICA 30, 60 or 120 mg/kg/day for 14 days. *In vitro*: ICA 60 μM	Fibrosis, TGF-β1/Smad signaling, collagen I/III and MMP-2/9↓; EF and FS↑.	(Jia et al. 2023)
	OGD/R-injured coronary microvascular endothelial cells	ICA 40 μM	Endothelial survival, migration and proliferation↑; TNF-α-dependent effects reported.	(Cao et al. 2026)
	OGD/R-treated H9C2 cardiomyoblasts	ICA 10 μM	ER-stress signaling, Drp1, NCOA4, Fe²⁺ and lipid peroxidation↓; FTH1↑.	(Zhong et al. 2024)
	I/R-treated H9C2 cells	ICA 5–15 μM	ROS, cytochrome c and caspases↓; HSP20, Bcl-2 and p-Akt↑.	(Ren et al. 2018)
	left anterior descending coronary artery ligation and reperfusion in rats	ICA 10 mg/kg	PI3K/Akt/eNOS and Bcl-2↑; Bax, caspase-3 and injury markers↓.	(Zhai et al. 2015)
	Mouse *in vivo* I/R, isolated rat hearts and cardiomyocyte simulated I/R	*In vivo*: ICA 60 mg/kg by gavage twice daily for 14 days. *Ex vivo*: 10 μM. *In vitro*: 4 μM	SIRT1, mitochondrial function and cardiac function↑; oxidative injury and apoptosis↓.	(Wu et al. 2018)
	Hypoxia/reoxygenation-treated H9C2 cells	ICA 2.5–20 μM	Fe²⁺, ACSL4, ROS and MDA↓; GPX4 and Nrf2/HO-1↑.	(Liu XJ et al. 2021)
Cerebral ischemia	MCAO rats	ICA 10 or 30 mg/kg by gavage twice daily for 15 days before MCAO	Infarct volume and neurological deficits↓; CypD, VDAC and Bax↓; Bcl-2↑.	(Zhou et al. 2024)
	MCAO mice	ICA or ICT 60 mg/kg	Apoptosis and extracellular-matrix markers↓.	(Wu et al. 2021)
	MCAO rats	ICA 60 mg/kg/day alone or combined with mild hypothermia	Inflammatory and apoptotic markers↓; combination with mild hypothermia produced greater changes.	(Dai et al. 2021)
	OGD/R-treated primary microglia	ICA 0.37–1.48 μM	IRE1/XBP1, NLRP3, caspase-1 and inflammatory cytokines↓.	(Mo et al. 2021)
	MCAO rats	ICA 10, 20 or 40 mg/kg	ER-stress signaling, TXNIP/NLRP3 and caspase-1↓.	(Zheng et al. 2022)
	MCAO rats	ICA 10 or 30 mg/kg	NF-κB-related inflammation↓; PPARα and PPARγ↑.	(Xiong et al. 2016)
	OGD/R-treated primary cortical neurons and microglia	ICA 0.37–1.48 μM	IRE1α/XBP1, CHOP, Bax and cleaved caspase-3↓; Bcl-2 and survival↑.	(Mo et al. 2020)
	MCAO rats	ICA 60 mg/kg/day intraperitoneally for 7 days	G3BP1 binding/oligomerization and stress-granule assembly↑; ischemic injury↓.	(Li L et al. 2026)
	OGD/R-treated primary neural cells	ICA 5–15 μM after OGD/R	ROS, LDH and intracellular Ca²⁺↓.	(Ning and Gao 2023)
	MCAO mice and OGD-treated primary cortical neurons	*In vivo*: ICA 50, 100 or 200 mg/kg. *In vitro*: ICA 0.15–9.6 μM	SIRT1, PGC-1α and neuronal survival↑.	(Zhu et al. 2010)
	Oxygen–glucose-deprived neuronal cells	ICA 0.1–9.6 μM	SIRT1 and neuronal survival↑.	(Wang et al. 2009)
	PC12 cells exposed to 2 h OGD and 24 h reperfusion	ICA 10 μM from 1 h before OGD through the 2 h OGD period	Viability and Bcl-2↑; Bax, cleaved caspase-3 and autophagy markers↓.	(Mo et al. 2016)
	PC12 cells exposed to 2 h OGD	ICA 0.1, 1 or 10 μM from 1 h before OGD through the OGD period	Viability↑; extracellular stress/injury markers↓.	(Mo et al. 2017)
	OGD-treated N2a neuronal cells	ICA 1–80 μM during OGD and reperfusion. Maximal viability at 10 μM	Apoptosis, inflammatory transcripts and PKM2↓.	(Chen et al. 2024)
	Global cerebral I/R mice and OGD/R-treated cortical neurons	*In vivo*: ICA 10, 30 or 100 mg/kg. *In vitro*: ICA 0.25, 0.5 or 1 mg/L, equivalent to approximately 0.37, 0.74 or 1.48 μM	Apoptosis, LDH, MDA and Ca²⁺↓; SOD and neuronal survival↑.	(Li et al. 2005)
	MCAO rats	Icariside II 10 or 30 mg/kg twice daily for 3 days before MCAO	Infarct volume and inflammation↓; PPARα/γ↑.	(Deng et al. 2016)
	MCAO mice	ICT 60 mg/kg intraperitoneally after ischemia	Apoptosis and senescence-associated proteins↓.	(Wu et al. 2022)
	MCAO rats	Icariside II (ICS) 20 mg/kg/day intraperitoneally for 7 days	TPI1 targeting, mitochondrial cristae organization and oxidative phosphorylation↑; neuroinflammation↓.	(Zhang et al. 2026)

### Endothelial dysfunction and phenotypic changes

Across endothelial models, ICA reduced inflammatory activation and leukocyte adhesion, improved NO-dependent vascular function, and modulated stress-response pathways. In ox-LDL-treated HUVECs, ICA reduced ICAM-1, VCAM-1, and E-selectin expression and monocyte adhesion (Hu et al. 2015). In ApoE−/− mice and LPC-treated HUVECs, ICA reduced PRMT I and ADMA, increased DDAH II expression and activity, and improved endothelium-dependent aortic relaxation (Xiao et al. 2015). A later study using ApoE−/− mice and LPC-treated HUVECs reported higher eNOS/NO, lower vascular ROS, and smaller aortic-root lesions after ICA treatment (Xiao et al. 2017). In homocysteine-treated endothelial cells, ICA reduced senescence and restored Akt, ERK, and eNOS phosphorylation (Xiao-Hong et al. 2013). In H_2_O_2_/DTT-treated HUVECs, 100 μM ICA increased cell viability and adhesion, increased SOD and GSH-Px activities, and reduced apoptosis and ER-stress markers including GRP78, ATF4, and eIF2α (Wang et al. 2019). In this study, significant protection against H_2_O_2_-induced injury was reported only at 100 μM among the ICA concentrations tested. The LPC-, homocysteine-, and ox-LDL-based endothelial experiments summarized here used concentrations within 0.1–40 μM. Testing GRP78, ATF4, and eIF2α at lower ICA concentrations would determine whether the ER-stress response is also detectable within this range. In diabetic vascular models, ICA reduced ER-stress markers and restored eNOS phosphorylation and vasorelaxation (Yao et al. 2021). In a second diabetic model, ICA reduced HMGB1 acetylation, nuclear translocation, and extracellular release together with RAGE/TLR4/NF-κB signaling. GPER inhibition prevented the ICA-induced increase in SIRT1, whereas SIRT1 inhibition or silencing restored HMGB1 translocation and inflammatory signaling (Yao et al. 2024).

In ox-LDL-induced endothelial-to-mesenchymal transition (EndMT), ICA restored CD31 and VE-cadherin and reduced α-SMA, S100A4, and endothelial cell migration. H19 knockdown attenuated these changes (Liu S et al. 2021). ICA also reduced apoptosis in ox-LDL-treated HUVECs, with increased Bcl-2 and decreased caspase-3 expression (Hu et al. 2018). Another study used ox-LDL-treated HUVECs and ApoE−/− mice to investigate TFEB, the autophagy–lysosome pathway, and ferroptosis-related injury. ICA promoted TFEB nuclear translocation and autophagosome–lysosome fusion while reducing intracellular Fe^2+^ accumulation, ROS, and lipid peroxidation. Blocking autophagosome formation with 3-methyladenine, disrupting autophagosome–lysosome fusion with bafilomycin A1, or silencing TRPML1 consistently attenuated the ICA-associated reductions in Fe^2+^ accumulation and lipid peroxidation. Microscale thermophoresis also detected ICA–TFEB binding (Wang X et al. 2023). The TFEB nuclear-translocation, autophagy-inhibition and TRPML1-silencing experiments support involvement of the autophagy–lysosome pathway in the ferroptosis-related changes. Current ferroptosis methodology emphasizes validated pharmacological modulators and genetic approaches when determining whether an observed phenotype reflects ferroptosis (Mishima et al. 2025). In plaque-stage ICA models, a more direct test could combine rescue with ferrostatin-1 or liproxstatin-1 and genetic perturbation of GPX4.

### Macrophage recruitment and foam-cell formation

In ApoE−/− mice, ICA reduced MOMA-2-positive macrophage accumulation in the aortic root and lowered arterial CX3CL1 and CX3CR1 expression. In LPS-stimulated RAW264.7 cells, ICA also reduced CX3CR1 expression and chemotaxis toward soluble CX3CL1 (Wang et al. 2016). These tissue and cell findings link lower lesional macrophage accumulation with reduced CX3CL1-directed chemotaxis. In THP-1-derived macrophages, ICA reduced ox-LDL binding and uptake, cholesteryl ester accumulation, and CD36 expression (Yang et al. 2015). CD36 overexpression partly restored ox-LDL uptake and foam-cell formation, anisomycin restored p38 phosphorylation and CD36 expression, and SR-BI silencing attenuated the reduction in intracellular lipid accumulation. ICA reduced CX3CL1/CX3CR1-dependent macrophage chemotaxis and lesional accumulation, while CD36 overexpression and SR-BI silencing modified its effects on ox-LDL uptake and foam-cell formation.

### VSMC remodeling and calcification

Across ox-LDL-based experiments, ICA reduced VSMC proliferation and migration, accompanied by changes in ERK1/2, ERBB4/AKT, PKCβI, and H19/HuR signaling. ICA at 10–40 μM reduced proliferation, inhibited G1/S cell-cycle progression, and lowered PCNA expression and ERK1/2 phosphorylation (Hu et al. 2016). Huang et al. combined an ApoE−/− atherosclerosis model with ox-LDL-treated human aortic VSMCs. In mice, ICA reduced vascular lipid accumulation and plaque formation. In VSMCs, ICA increased apoptosis and reduced proliferation and migration, while miR-205-5p silencing restored ERBB4/AKT signaling and attenuated the effects of ICA on proliferation and migration (Huang et al. 2023). Pharmacological activation of PKC attenuated ICA-associated reductions in PKCβI phosphorylation, cyclin D1, and MMP-9, while PKCβ inhibition enhanced these changes (Zhang et al. 2021). A later H19 study found that ICA reduced cyclin D1 and MMP-9 expression together with VSMC proliferation and migration, and H19 knockdown attenuated these effects (Zhang et al. 2025). ICA at 0.30 or 0.59 μM (0.2 or 0.4 mg/L) increased early apoptosis and GRP78 mRNA in homocysteine-treated VSMCs (Shen and He 2009). VSMC apoptosis is relevant to plaque stability because fibrous-cap VSMCs are an important source of collagen, and their loss can promote cap thinning and necrotic-core formation (Li et al. 2022). In the Huang study, ICA increased apoptosis in cultured VSMCs, whereas the mouse experiment quantified lipid accumulation and plaque formation. Measuring fibrous-cap thickness, collagen content, and the spatial distribution of apoptotic VSMCs would determine whether the increase in VSMC apoptosis observed *in vitro* also involves fibrous-cap VSMCs *in vivo*. The increase in VSMC apoptosis contrasts with the reduced apoptosis reported in endothelial injury models, although these responses have not been compared under matched conditions or within the same atherosclerotic plaque model. An earlier study with icaritin (ICT), an icariin-related metabolite, provides additional *in vivo* evidence on VSMC distribution and plaque collagen. In cholesterol-fed rabbits, ICT reduced lesional macrophage accumulation and aortic MMP-1 expression and activity, increased collagen protein in atherosclerotic lesions, and shifted SMC distribution toward the fibrous-cap region without increasing total SMC abundance (Zhang et al. 2016).

ICA also reduced osteogenic conversion and mineral deposition in VSMCs. In rat thoracic-aortic VSMCs exposed to high glucose and H_2_O_2_, ICA reduced alkaline phosphatase activity and calcium deposition, lowered osteocalcin, osteopontin, Runx2, and collagen I, restored α-SMA, and reduced ATF4 and eIF2α phosphorylation (Bai et al. 2021). In β-glycerophosphate-treated VSMCs, ICA reduced alkaline phosphatase activity, Runx2 expression, and mineral deposition while increasing p38 phosphorylation. SB203580 partly restored Runx2 expression, alkaline phosphatase activity, and calcification, while ERα antagonism attenuated p38 activation and the anti-calcific response (He et al. 2023). In hypercholesterolemic rats, aortic p38 phosphorylation decreased as circulating lipids, vascular inflammation, and lesion burden improved (Hu et al. 2016). The pharmacological intervention in calcifying VSMCs supports a functional role for p38 in the anti-calcific effect of ICA. The reduction in whole-aorta p38 phosphorylation was measured together with improvements in hypercholesterolemia-associated vascular injury and was not subjected to an equivalent p38 intervention.

### Lipid metabolism and PPAR signaling

Cumulative LDL-C exposure and severe hypercholesterolemia are strongly associated with ASCVD risk (Domanski et al. 2020; Lee et al. 2022). *In vivo*, lower lesion burden often coincided with lower circulating lipid levels. High-cholesterol-fed rats showed reductions in total cholesterol, triglycerides, and LDL-C together with less aortic lipid deposition (Hu Y et al. 2016). Concurrent reductions in circulating lipids and lesion burden were also reported in ApoE−/− models (Zhang et al. 2021; Huang et al. 2023; Wang X et al. 2023) and in the eNOS study (Xiao et al. 2017). Under a fixed extracellular ox-LDL challenge, however, ICA still reduced endothelial adhesion (Hu et al. 2015), macrophage ox-LDL binding and uptake (Yang et al. 2015), and VSMC proliferation and migration (Huang et al. 2023). Because extracellular ox-LDL was held constant, these cell experiments isolate vascular-cell responses from changes in the external lipid stimulus. Studies in skeletal muscle and liver provide mechanistic context for the systemic lipid changes observed *in vivo*. In C2C12 myotubes, AMPK inhibition or silencing blocked ICA-induced changes in PGC-1α and FNDC5 (Chen SQ et al. 2019). In high-fat-diet mice, ICA altered hepatic AMPKα1, PGC-1α, ACC, fatty-acid oxidation, and lipogenic proteins and reduced hepatic lipid accumulation (Lin et al. 2021). AMPK can regulate SREBP-dependent lipogenic transcription (Li et al. 2011; Zou et al. 2025), consistent with the changes in AMPK, ACC, and lipogenic proteins observed in this model. The reduction in lesion burden observed *in vivo* may therefore involve both systemic lipid lowering and vascular-cell responses that remain detectable under fixed ox-LDL exposure.

PPAR signaling has been examined in diabetes-related endothelial dysfunction, adipocyte differentiation, and cerebral ischemia. In diabetic vascular models, ICA promoted PPARα nuclear translocation, and GW6471 attenuated the reduction in ER-stress markers and the recovery of eNOS phosphorylation and vascular relaxation (Yao et al. 2021). In 3T3-L1 cells, ICA reduced PPARγ, C/EBPα, SREBP-1c, and intracellular lipid accumulation (Han et al. 2016), consistent with suppression of adipogenic differentiation. In cerebral ischemia, ICA increased PPARα and PPARγ while reducing IL-1β, TGF-β1, and NF-κB-related signaling (Xiong et al. 2016). The opposite PPARγ responses are consistent with its different roles in adipogenic and inflammatory programs. PPARγ activity also depends on cofactor recruitment and post-translational modifications, including phosphorylation, acetylation, and SUMOylation (Pascual et al. 2005; Montaigne et al. 2021). ICA may therefore affect PPARγ differently across tissues. If these tissue differences are functionally important, tissue-targeted delivery may become relevant to the development of ICA-based therapies.

### RNA-mediated regulation across vascular cell types

Aortic RNA profiling in ICA-treated ApoE−/− mice identified changes in both coding and non-coding transcripts. An mRNA–miRNA analysis identified altered miRNAs and linked the resulting regulatory network to PI3K/Akt, Ras, ErbB and VEGF-related pathways (Zhang et al. 2018), whereas lncRNA–mRNA profiling identified 1,512 differentially expressed lncRNAs and 2,059 mRNAs (Zhang et al. 2019). A 2023 study combined a new miRNA-chip analysis with functional testing of miR-205-5p. Silencing miR-205-5p attenuated the inhibition of VSMC proliferation and migration by ICA and reversed the associated changes in ERBB4/AKT signaling (Huang et al. 2023). Mechanistic studies of H19 identified different post-transcriptional interactions in endothelial cells and VSMCs. In ox-LDL-treated endothelial cells, H19 knockdown weakened the inhibition of EndMT by ICA, and perturbation of miR-148b-3p and ELF5 supported the H19–miR-148b-3p–ELF5 pathway (Liu S et al. 2021). In VSMCs, H19 bound HuR and reduced HuR association with cyclin D1 and MMP-9 mRNAs, lowering their stability and contributing to the inhibition of proliferation and migration by ICA (Zhang et al. 2025).

H19 regulation also differed across the endothelial studies. In the ICA model, ox-LDL reduced H19 expression, whereas ICA restored H19 together with a reduction in EndMT (Liu S et al. 2021). In two other endothelial studies, H19 increased after ox-LDL exposure (Tang et al. 2024; Hu et al. 2026). METTL3-dependent m6A modification and IGF2BP2 binding increased H19 stability in one model, and H19 re-expression after METTL3 knockdown restored inflammatory and pyroptotic responses (Tang et al. 2024). In another model, H19 regulated let-7a–ITGB3 signaling, and H19 silencing reduced adhesion molecule expression and atherosclerotic lesion formation (Hu et al. 2026). H19 therefore participates in different post-transcriptional networks in endothelial cells and VSMCs, including H19–miR-148b-3p–ELF5 in endothelial cells and H19–HuR in VSMCs (Liu S et al. 2021; Zhang et al. 2025). Differences in basal H19 abundance between these cell types may also influence their responses to ICA. ICA may further alter which RNA or protein partners are engaged by H19 in each cell type. Comparing H19 abundance, subcellular distribution, and binding partners under the same ICA exposure would help determine whether these responses are coordinated within the plaque. The principal ICA-related mechanisms discussed in Sections ‘Endothelial dysfunction and phenotypic changes’–’RNA-mediated regulation across vascular cell types’ are summarized in Figure 3.

**Figure 3.**
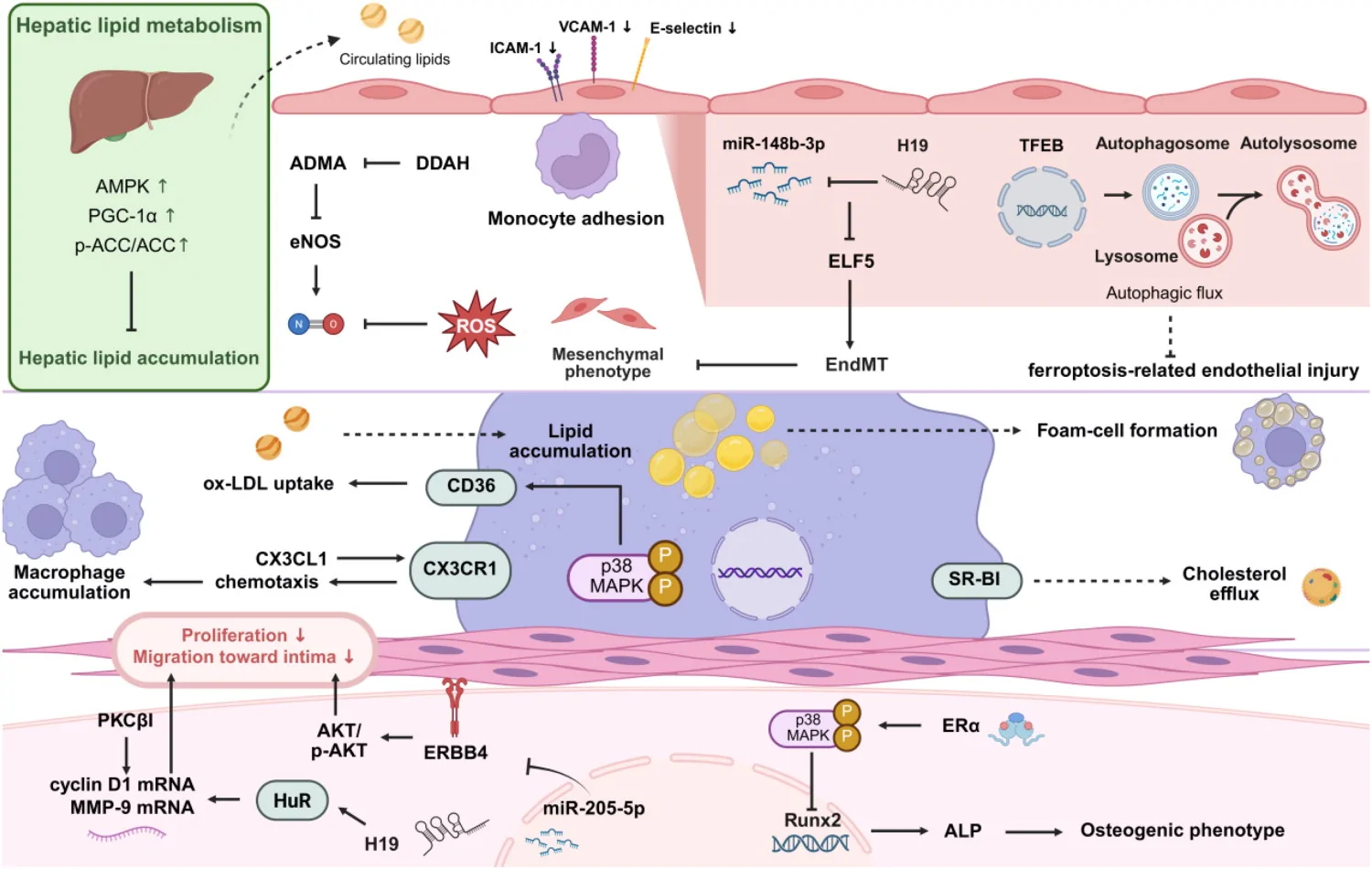
Icariin-related mechanisms in atherosclerotic plaque biology. Icariin-associated changes in hepatic lipid metabolism and circulating lipids have been reported *in vivo* (Hu Y et al. 2016; Xiao et al. 2017; Lin et al. 2021; Zhang et al. 2021; Huang et al. 2023; Wang X et al. 2023). Endothelial pathways involve adhesion molecule expression and monocyte adhesion (Hu et al. 2015), DDAH–ADMA–eNOS/NO signaling and oxidative stress (Xiao et al. 2015, 2017), H19–miR-148b-3p–ELF5-mediated endothelial-to-mesenchymal transition (EndMT) (Liu S et al. 2021), and TFEB-associated autophagy and ferroptosis-related injury (Wang X et al. 2023). In macrophages, the figure includes CX3CL1–CX3CR1-dependent chemotaxis and lesional macrophage accumulation (Wang et al. 2016), together with p38 MAPK–CD36- and SR-BI-related lipid handling and foam-cell formation (Yang et al. 2015). In vascular smooth muscle cells (VSMCs), reported pathways include miR-205-5p–ERBB4–AKT (Huang et al. 2023), PKCβI–cyclin D1/MMP-9 (Zhang et al. 2021), H19–HuR (Zhang et al. 2025), and ERα–p38 MAPK-dependent osteogenic differentiation (He et al. 2023). Solid lines indicate experimentally examined relationships, dashed lines indicate indirect or proposed connections, and blunt-ended lines indicate inhibition. Upward and downward arrows indicate increases and decreases, respectively, after ICA treatment relative to the corresponding model control.

## Post-ischemic protection

Post-ischemic evidence includes post-MI remodeling, acute myocardial I/R injury, and cerebral ischemic injury. Post-MI experiments characterize fibrosis and ventricular remodeling after infarction. Myocardial I/R experiments capture acute reperfusion injury, whereas cerebral ischemia studies relate cellular stress responses to infarct burden and neurological outcomes.

### Post-myocardial infarction remodeling and repair

Across post-MI studies, ICA was associated with reduced myocardial fibrosis and cardiomyocyte apoptosis, accompanied by improved ventricular function. After permanent coronary ligation, ICA reduced collagen I and III, CD147, and MMP-9, increased TIMP-1, and reduced cardiomyocyte apoptosis. Exogenous CD147 partly reversed the changes in collagen deposition, apoptosis, and ventricular function (Shi et al. 2018). In a separate left anterior descending coronary artery ligation model, ICA reduced collagen I and III, MMP-2, MMP-9, TGF-β1, and Smad2/3 phosphorylation. These changes were accompanied by less myocardial fibrosis and better cardiac function (Jia et al. 2023). In the same study, ICA also reduced TGF-β1 and Smad2/3 phosphorylation in IL-4-treated RAW264.7 macrophages. Macrophage Smad3 has independently been shown to regulate phagocytosis and inflammatory responses after myocardial infarction (Chen B et al. 2019). A separate MI study extended the immune analysis to adaptive lymphocyte populations. ICA reduced CD8^+^ cytotoxic T cells and CD3^+^CD4^+^IFN-γ^+^ Th1 cells, increased CD3^+^CD4^+^Foxp3^+^ regulatory T cells, and was accompanied by lower inflammatory cytokines, less fibrosis and apoptosis, and improved left-ventricular function (Sai et al. 2022).

A separate study focused on coronary microvascular endothelial repair after myocardial infarction. In OGD/R-injured coronary microvascular endothelial cells, 40 μM ICA increased cell survival, migration, and proliferation, while TNF neutralization with infliximab attenuated these effects. Bio-layer interferometry detected binding between ICA and TNF-α (Cao et al. 2026). The attenuation produced by infliximab directly tested TNF dependence of the ICA response in CMECs. The animal arm evaluated TNF knockdown without ICA treatment and showed larger infarcts, further deterioration of cardiac function, and lower microvascular density and endothelial regeneration.

### Cardiac ischemia–reperfusion injury

Cardiac I/R studies primarily assessed acute reperfusion-associated injury, with infarct size, apoptosis, oxidative stress, and mitochondrial function as major endpoints. The PI3K inhibitor LY294002 attenuated ICA-associated protection in an *in vivo* rat I/R model (Meng et al. 2015), Langendorff-perfused rat hearts (Ke et al. 2015), and a second *in vivo* rat I/R model (Zhai et al. 2015). In these studies, LY294002 attenuated ICA-associated reductions in infarct size or apoptosis and blunted Akt or Akt/eNOS phosphorylation. In Langendorff-perfused hearts, 5 or 10 μM ICA improved cardiac function, reduced CK-MB, LDH, MDA, and apoptosis, and increased SOD activity. In an H9C2 I/R model, HSP20 overexpression increased cell proliferation and reduced apoptosis and ROS, changes that paralleled those observed with ICA (Ren et al. 2018). SIRT1 inhibition or silencing attenuated preservation of mitochondrial membrane potential and increased FOXO1 acetylation and cytochrome c release (Wu et al. 2018). Later H9C2 studies examined ferroptosis-related mechanisms within acute reperfusion injury.

In H9C2 cells exposed to hypoxia/reoxygenation, ICA increased cell viability and reduced LDH, Fe^2+^, ACSL4, ROS and MDA while increasing GPX4, Nrf2 and HO-1. Erastin reversed the ICA-associated inhibition of ferroptosis. In ICA-treated hypoxia/reoxygenation cells, the HO-1 inhibitor ZnPP decreased cell viability and GPX4 and increased LDH, Fe^2+^ and ACSL4 (Liu XJ et al. 2021). In a separate OGD/R study, ICA reduced IRE1/JNK activation, Drp1 mitochondrial translocation, NCOA4 expression, Fe^2+^ accumulation, and lipid peroxidation and increased FTH1 (Zhong et al. 2024). Perturbation of IRE1/JNK and Drp1 altered mitochondrial fission, mitophagy, and ferroptosis-related changes. Erastin and ZnPP provided pharmacological tests of ferroptosis and HO-1 in the hypoxia/reoxygenation model. The OGD/R study extended the analysis to IRE1/JNK and Drp1, linking ER stress to mitochondrial fission, mitophagy, and ferroptosis-related changes. Testing Drp1 perturbation together with ferroptosis and apoptosis inhibitors in the same OGD/R model, with cell viability, lipid peroxidation, and mitochondrial morphology measured under each intervention, would clarify the relation of Drp1-dependent mitochondrial fission to ferroptotic and apoptotic injury.

### Cerebral ischemic injury

Detailed mechanistic studies after cerebral ischemia have focused on IRE1/PERK-linked ER-stress signaling and G3BP1-dependent stress-granule assembly. In middle cerebral artery occlusion (MCAO) rats, ICA reduced IRE1α and PERK activation together with XBP1s, TXNIP, NLRP3, caspase-1, and IL-1β and improved cerebral edema and neurological deficits (Zheng et al. 2022). The proposed interactions of ICA with GRP78, IRE1α, and PERK in this study were derived from molecular docking. In OGD/R-treated primary microglia, ICA reduced XBP1s, NLRP3, caspase-1, IL-1β, IL-6, and TNF-α, while IRE1 overexpression restored IRE1/XBP1 signaling and attenuated the anti-inflammatory changes (Mo et al. 2021). In neuronal OGD/R, reduced IRE1α/XBP1 signaling was accompanied by lower CHOP, Bax, and cleaved caspase-3 and higher Bcl-2 (Mo et al. 2020). These experiments support a role for IRE1/XBP1 signaling in the anti-inflammatory and anti-apoptotic effects associated with ICA. A separate MCAO study identified G3BP1-dependent stress-granule assembly as another mechanistically tested pathway. ICA bound the NTF2L domain of G3BP1, promoted G3BP1 oligomerization and stress-granule assembly, and increased IGF2BP1 recruitment. G3BP1 depletion reduced stress-granule formation and attenuated the effects of ICA on infarct volume and neurological function (Li L et al. 2026).

Zhou et al. assessed mitochondrial permeability transition and oxidative injury in MCAO rats treated with parent ICA and H_2_O_2_-treated PC-12 cells treated with icaritin. *In vivo*, ICA reduced infarct volume and neurological deficits, decreased CypD, VDAC, and Bax, and increased Bcl-2. *In vitro*, icaritin reduced ROS and mPTP opening, preserved mitochondrial membrane potential, and its protective effect was reversed by lonidamine (Zhou et al. 2024). A separate OGD/R neural-cell study using parent ICA reported lower ROS, LDH release, and intracellular Ca^2+^ after treatment (Ning and Gao 2023). An earlier neuronal study directly tested SIRT1 involvement, showing that ICA increased SIRT1 expression and neuronal survival after OGD and that SIRT1 inhibition attenuated this protection (Wang et al. 2009). In a later cerebral ischemia study, SIRT1 inhibition or PGC-1α silencing attenuated ICA-associated neuroprotection (Zhu et al. 2010). The direct mPTP-opening and mitochondrial-membrane-potential data in Zhou et al. therefore derive from icaritin-treated PC-12 cells, whereas the SIRT1/PGC-1α perturbation studies used parent ICA. The principal mechanisms reported in myocardial and cerebral ischemic injury models are summarized in Figure 4.

**Figure 4.**
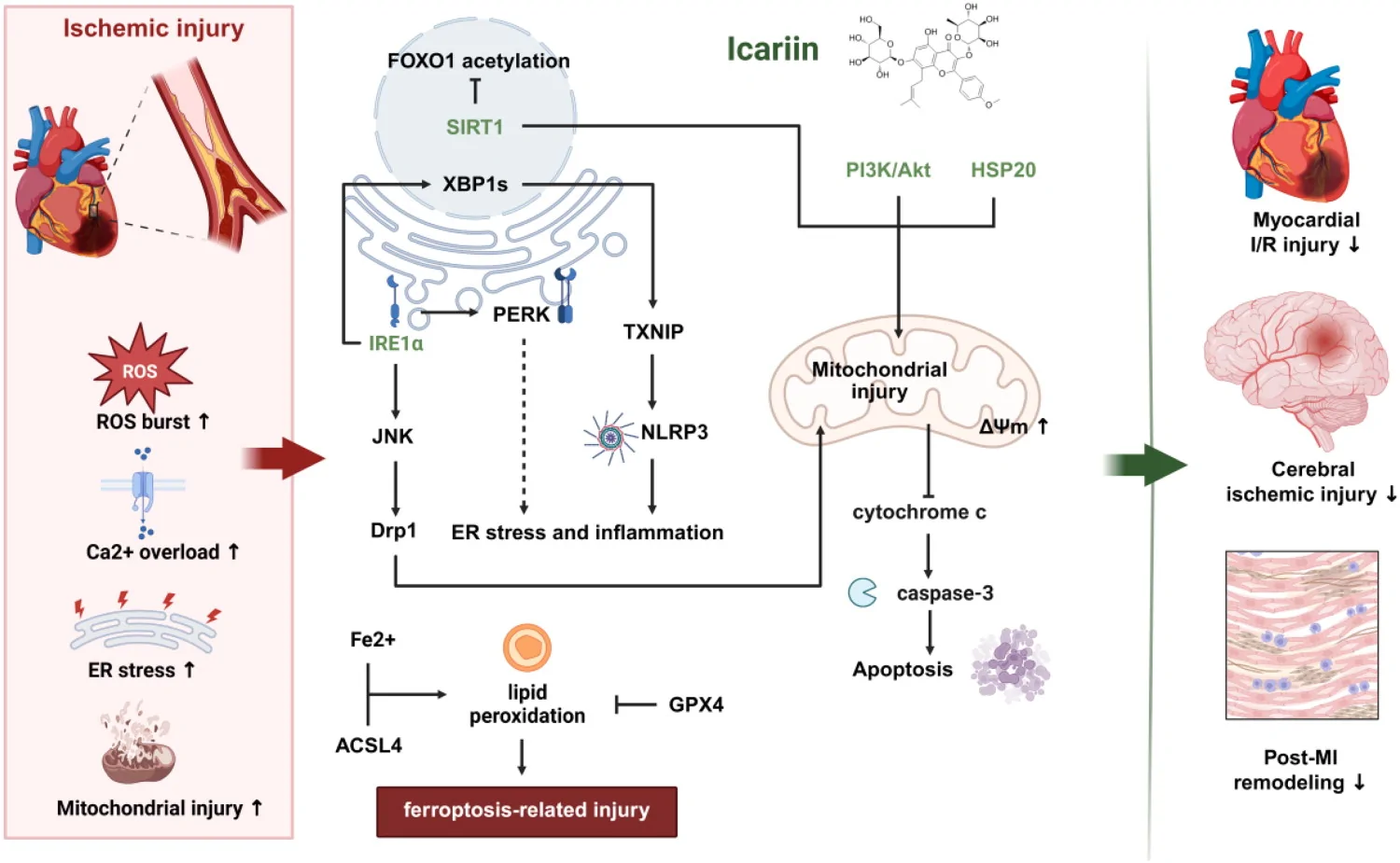
Icariin-related mechanisms in myocardial and cerebral ischemic injury. In cardiac ischemia–reperfusion models, PI3K–Akt signaling (Ke et al. 2015; Meng et al. 2015; Zhai et al. 2015), HSP20 (Ren et al. 2018), and SIRT1-dependent regulation of FOXO1 acetylation and mitochondrial injury (Wu et al. 2018) were implicated in the protective effects of icariin. The IRE1α–JNK–Drp1 pathway and ferroptosis-related changes were examined in cardiomyocytes subjected to oxygen–glucose deprivation/reoxygenation (Zhong et al. 2024), whereas a separate hypoxia/reoxygenation model assessed Fe^2+^ accumulation, ACSL4, GPX4, and lipid peroxidation (Liu XJ et al. 2021). In cerebral ischemia models, ER-stress signaling involved IRE1α, PERK, and XBP1s, with TXNIP–NLRP3-related inflammasome responses (Mo et al. 2020, 2021; Zheng et al. 2022) and additional evidence for mitochondrial and oxidative injury (Ning and Gao 2023; Zhou et al. 2024). Across these models, icariin treatment was associated with reduced myocardial I/R injury (Ke et al. 2015; Meng et al. 2015; Zhai et al. 2015; Ren et al. 2018; Wu et al. 2018; Liu XJ et al. 2021; Zhong et al. 2024) and cerebral ischemic injury (Mo et al. 2020, 2021; Zheng et al. 2022; Ning and Gao 2023; Zhou et al. 2024; Li L et al. 2026), while separate myocardial infarction studies reported attenuation of post-MI remodeling (Shi et al. 2018; Sai et al. 2022; Jia et al. 2023). The pathways shown derive from separate experimental systems and should not be interpreted as a single continuous signaling Cascade. Green labels identify signaling nodes whose involvement in ICA-associated protection was examined in the cited studies. Blunt-ended lines indicate inhibition, and dashed lines indicate indirect or proposed connections. Within the ischemic-injury panel, upward arrows indicate changes induced by ischemic injury, whereas downward arrows beside the terminal outcomes indicate reductions associated with ICA treatment.

## Translational limitations of icariin

### Pharmacokinetics, metabolism and human exposure

ICA has low aqueous solubility, poor membrane permeability and slow dissolution, and oral administration generally produces low plasma and tissue exposure (Szabó et al. 2022). It undergoes deglycosylation, dehydrogenation, hydroxylation, glucuronidation and demethylation, with multiple metabolites detected in feces, bile and urine (Sun et al. 2016). Icaritin is also poorly soluble, is absorbed incompletely after oral administration and undergoes extensive metabolism (Zhang et al. 2017). It is distributed mainly to the liver and is glucuronidated by UGT1A3, UGT1A9 and UGT2B7, followed by biliary, fecal and urinary excretion (Zhang 2016; Wang et al. 2018). These pharmacokinetic characteristics make administered dose an incomplete indicator of the concentrations of parent ICA and related metabolites reaching vascular, myocardial or cerebral tissue.

Human studies provide direct evidence of this exposure limitation. In a randomized, double-blind, placebo-controlled study of 24 healthy adults, an ICA-containing commercial preparation standardized to 20% ICA was given at target ICA doses of 100–1,680 mg/day for 5 days. Almost all plasma ICA concentrations were below the 1 ng/mL lower limit of quantification, and two participants receiving 1,680 mg/day discontinued treatment because of gastrointestinal symptoms (Brown et al. 2019). In 30 healthy men given a single dose of standardized Epimedium prenylflavonoids, icariside II and desmethylicaritin were the principal circulating compounds, whereas parent ICA remained below the detection limit (Teo et al. 2019). Similar findings were obtained in 58 postmenopausal women receiving Epimedium prenylflavonoids at 740 mg/day for 6 weeks. No significant adverse symptoms or changes in hepatic, renal or hematological indices were reported, while desmethylicaritin, icaritin and icariside II were detected in serum and parent ICA remained below the detection limit (Yong et al. 2021). These studies used conventional oral preparations and did not measure parent ICA or metabolite concentrations in cardiovascular diseased tissue.

### Toxicology and drug-interaction evidence

Safety studies have examined parent ICA, Epimedium extracts and individual downstream prenylflavonoids under different experimental conditions. Direct exposure of zebrafish embryos to parent ICA at 10 or 40 μM reduced hatching and body length and produced swim-bladder abnormalities, while 40 μM ICA also altered thyroid-hormone indices and thyroid-related gene expression (Wu et al. 2023). In studies of Epimedium preparations, comparison of four extracts and 11 shared constituents identified icariside I and sagittatoside A as the constituents most closely associated with extract cytotoxicity; the effects involved oxidative stress and mitochondrial injury (Zhang et al. 2020). In mice receiving an Epimedii Folium water extract for 14 weeks, cholestatic liver injury was most evident at the high dose and was accompanied by altered bile-acid transport (Zhao et al. 2022). Recent studies further distinguish individual prenylflavonoids. Icariside II showed the greatest hepatotoxicity among five flavonoids tested in a liver microfluidic-organ-chip and hepatocyte model (Cui et al. 2026), whereas icariside I produced HK-2 cytotoxicity and renal injury in mice accompanied by oxidative stress and altered PI3K/AKT/Nrf2/HO-1 signaling (Wang et al. 2026). Pharmacokinetic comparison of potentially hepatotoxic prenylflavonoids from Epimedium extract showed that icaritin had the highest systemic exposure and greater accumulation potential (Peng et al. 2026).

Drug-interaction data remain preclinical. ICA inhibited OATP1B3- and OATP2B1-mediated substrate uptake *in vitro* (Li et al. 2014), while ICA and its intestinal metabolites showed different inhibitory profiles against human UGT isoforms (Cao et al. 2012). In mice receiving oral ICA at 40–320 mg/kg/day for 7 days, hepatic UGT activity and Cyp2b10 and Cyp3a11 expression were not reduced, although Cyp4a14 and several Ugt2b transcripts changed at selected doses (Xu et al. 2014). More recently, icaritin inhibited multiple human UGT isoforms *in vitro*, with kinetic analysis of UGT1A1, UGT1A3 and UGT1A9 and confirmation of UGT1A1 and UGT1A9 inhibition in human liver microsomes (Rong et al. 2024). Direct coadministration studies evaluating ICA with statins, antiplatelet agents, anticoagulants or other standard ASCVD drugs were not identified.

### Formulation and translational requirements

Ding et al. formulated total flavonoids of *Epimedium* brevicornu as nanosuspensions, cyclodextrin inclusion complexes and solid dispersions and measured several constituent flavonoids in rat plasma. Relative to crude total flavonoids, the solid-dispersion formulation increased the relative bioavailability of ICA to 416% (Ding et al. 2025). The 416% value reflects plasma ICA exposure after administration of a multicomponent *Epimedium* flavonoid preparation; cardiovascular tissue exposure was not measured.

Kamal et al. developed ICA-loaded protein-free lipid nanoparticles mimicking high-density lipoprotein (HDA) and low-density lipoprotein (LDE). Both formulations showed high encapsulation efficiency and good serum stability. In rats with myocardial infarction, HDA accumulated more strongly in infarcted myocardium and produced greater cardiac protection than LDE (Kamal et al. 2025). These biodistribution data concern infarcted myocardium; vascular-wall or plaque distribution was not examined. Polymeric micelles, nanosuspensions, hydrogels and liposomes have also been examined for ICA or related compounds (Hui-Rong et al. 2020; Wang et al. 2020; Xu et al. 2020; Tang et al. 2021), but most of these studies were conducted outside cardiovascular disease models. The main translational limitations and priorities for further study are summarized in Table 2.

**Table 2.** Translational considerations for icariin in ASCVD based on preclinical pharmacology, formulation and safety evidence.

Area	Current evidence	Key limitations	Further studies	Representative references
Atherosclerosis	Endothelial dysfunction; macrophage recruitment; foam-cell formation; VSMC remodeling; plaque burden	Limited plaque composition and stability data	Measure fibrous-cap thickness, necrotic-core area, and plaque exposure to parent ICA and major metabolites, and relate exposure to plaque composition.	(Zhang et al. 2013; Yang et al. 2015; Wang et al. 2016; Xiao et al. 2017; Liu S et al. 2021; Zhang et al. 2021; Huang et al. 2023; Wang X et al. 2023)
Myocardial infarction and I/R	MI, I/R and H/R models; apoptosis; ferroptosis; mitochondrial injury; ventricular remodeling	Heterogeneous treatment windows; limited long-term functional data	Test treatment initiated after ischemia or reperfusion, quantify infarct size and long-term remodeling and function, and evaluate ICA with standard cardiovascular therapy.	(Zhai et al. 2015; Ren et al. 2018; Shi et al. 2018; Liu XJ et al. 2021; Sai et al. 2022; Jia et al. 2023; Zhong et al. 2024)
Cerebral ischemia	MCAO and OGD/R models; mitochondrial and ER stress; inflammation; apoptosis	Parent and metabolite evidence not consistently separated; brain exposure unknown	Test post-stroke treatment, measure brain exposure to parent ICA and major metabolites, assess neurological recovery, and evaluate ICA alongside reperfusion therapy.	(Wang et al. 2009; Zhu et al. 2010; Deng et al. 2016; Xiong et al. 2016; Mo et al. 2020, 2021; Wu et al. 2021; Zheng et al. 2022; Ning and Gao 2023; Zhou et al. 2024; Li L et al. 2026)
Formulation and delivery	Micelles; nanosuspensions; intranasal gels; liposomes; lipoprotein-inspired carriers	Most systems tested outside ASCVD; no plaque biodistribution data	Measure plasma and target-tissue pharmacokinetics, plaque or myocardial distribution, and cellular uptake, and assess repeat-dose safety of the final formulation.	(Hui-Rong et al. 2020; Wang et al. 2020; Xu et al. 2020; Tang et al. 2021; Ding et al. 2025; Kamal et al. 2025)
Pharmacokinetics and safety	Low oral parent-ICA exposure; circulating metabolites; toxicology signals; UGT/transporter interactions	Limited repeat-dose safety data; no clinical ASCVD drug-interaction studies	Define steady-state pharmacokinetics of parent ICA and major metabolites, assess repeat-dose safety, and test interactions with standard ASCVD drugs.	(Brown et al. 2019; Rong et al. 2024; Cui et al. 2026)

Cardiovascular development of these formulations requires exposure measurements to be incorporated into the same experiments used to assess efficacy and safety. Plasma pharmacokinetics should distinguish parent ICA from major metabolites, while tissue measurements should match the intended indication, including vascular wall or plaque for atherosclerosis and myocardium for post-ischemic cardiac applications. In atherosclerosis models, tissue exposure should also be related to plaque composition and stability rather than lesion area alone. Post-MI and myocardial I/R studies should determine whether efficacy is retained when treatment begins after ischemia or reperfusion and when ICA is administered with contemporary reperfusion, lipid-lowering or antithrombotic therapy. Repeat-dose toxicology and drug-interaction studies should use the same formulation and an exposure range comparable with that associated with efficacy. The principal unresolved priorities are tissue exposure of parent ICA and major metabolites, more complete characterization of plaque structure and composition, efficacy with clinically relevant treatment timing and background therapy, and repeat-dose safety and drug interactions. The distribution of the available evidence across study types and settings is summarized in Figure 5.

**Figure 5.**
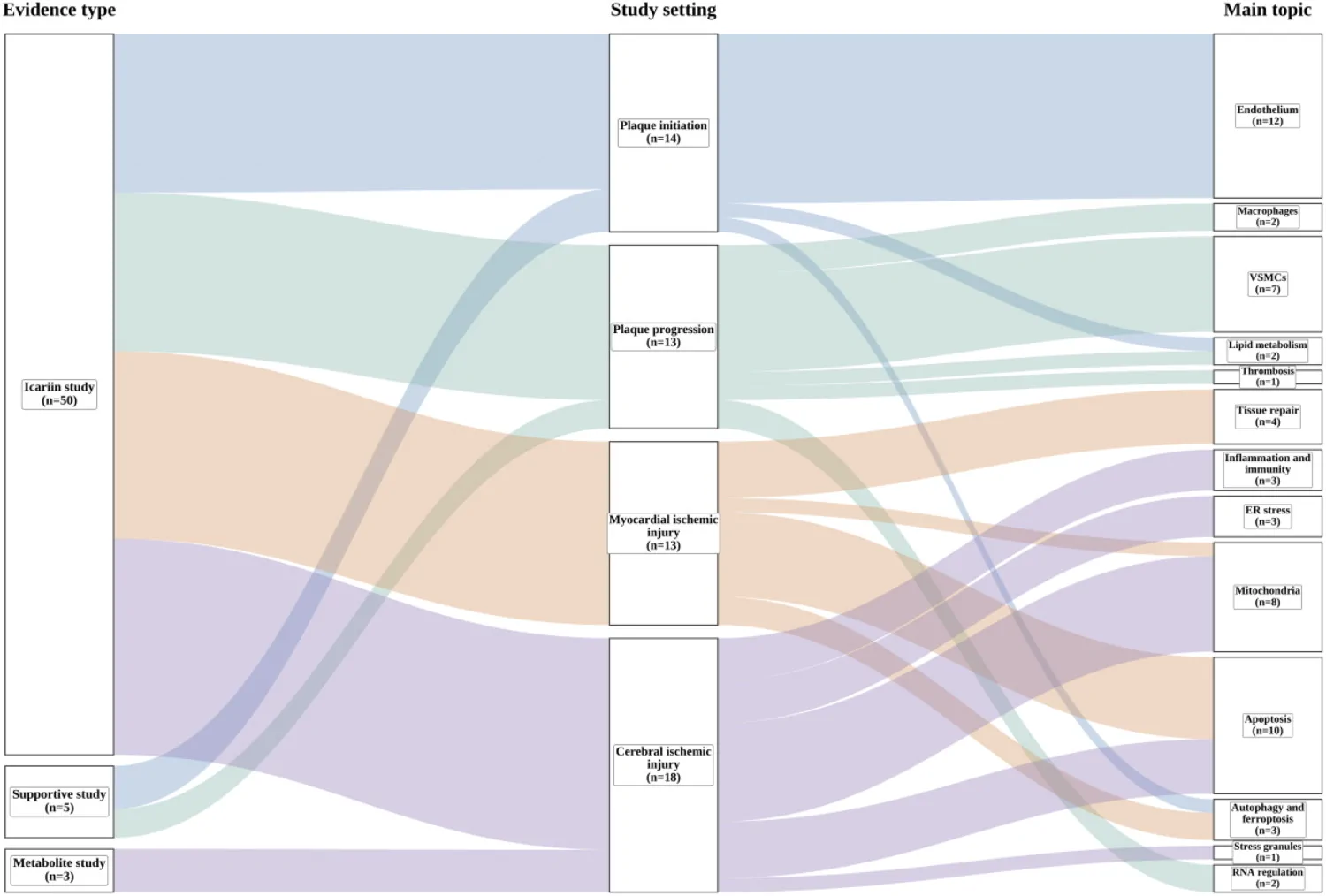
Evidence map of the available icariin literature by evidence type, study setting and main topic. Node values (n) and flow widths represent study counts. Labels below the diagram summarize the principal translational priorities.

## Conclusions

Experimental studies show that ICA reduces endothelial inflammatory activation, macrophage lipid uptake, VSMC proliferation and migration, and atherosclerotic lesion burden, while post-MI and cerebral ischemia models report reductions in fibrosis and ischemic tissue injury. Further atherosclerosis studies should measure parent ICA and active metabolites in diseased tissues together with plaque composition and stability. Post-ischemic studies should test treatment initiated after ischemia or reperfusion and in the presence of contemporary background therapy. As formulations increase systemic or tissue exposure, chronic safety and interactions with standard ASCVD drugs should be evaluated in parallel.

## Supplementary Material

补充材料2.docx

Supplementary_File_1_PRISMA_2020_checklist.docx

NPHB-2026-0659.R1_Supplementary_File_3_SANRA.docx

## Data Availability

There are no data associated with this research.
