# Supplementary material for: Protective effects of icariin in atherosclerotic cardiovascular disease: mechanistic basis, post-ischemic protection and translational limitations
Source: Pharm Biol. 2026 Oct 10;64(1):1066–86. doi: 10.1080/13880209.2026.2740872 (PMC13656681; doi:10.1080/13880209.2026.2740872)
Supplement: Supplementary file 2 [file IPHB_A_2740872_SM1047.docx]

| **Section and Topic** | **Item #** | **Checklist item** | **Location where item is reported** |
| --- | --- | --- | --- |
| **TITLE** | | |  |
| Title | 1 | Identify the report as a systematic review. | Title page. The manuscript is submitted as a Review with a structured literature search rather than as a systematic review. |
| **ABSTRACT** | | |  |
| Abstract | 2 | See the PRISMA 2020 for Abstracts checklist. | Abstract. |
| **INTRODUCTION** | | |  |
| Rationale | 3 | Describe the rationale for the review in the context of existing knowledge. | Introduction. |
| Objectives | 4 | Provide an explicit statement of the objective(s) or question(s) the review addresses. | Introduction; final paragraph states the purpose and scope of the review. |
| **METHODS** | | |  |
| Eligibility criteria | 5 | Specify the inclusion and exclusion criteria for the review and how studies were grouped for the syntheses. | Materials and methods; eligibility criteria and exclusion criteria. |
| Information sources | 6 | Specify all databases, registers, websites, organisations, reference lists and other sources searched or consulted to identify studies. Specify the date when each source was last searched or consulted. | Materials and methods; PubMed and Web of Science Core Collection, searched up to 4 May 2026. |
| Search strategy | 7 | Present the full search strategies for all databases, registers and websites, including any filters and limits used. | Materials and methods; full PubMed and Web of Science search strategies are reported. |
| Selection process | 8 | Specify the methods used to decide whether a study met the inclusion criteria of the review, including how many reviewers screened each record and each report retrieved, whether they worked independently, and if applicable, details of automation tools used in the process. | Materials and methods; title/abstract screening, full-text reassessment when required and predefined criteria. Fig. 1 shows the selection process. |
| Data collection process | 9 | Specify the methods used to collect data from reports, including how many reviewers collected data from each report, whether they worked independently, any processes for obtaining or confirming data from study investigators, and if applicable, details of automation tools used in the process. | Materials and methods; information was narratively extracted according to model type, intervention, pharmacological readouts and translational constraints. |
| Data items | 10a | List and define all outcomes for which data were sought. Specify whether all results that were compatible with each outcome domain in each study were sought (e.g. for all measures, time points, analyses), and if not, the methods used to decide which results to collect. | Materials and methods; data sought included ASCVD-related vascular, myocardial ischemic and cerebral ischemic models, pharmacological readouts and translational limitations. |
|  | 10b | List and define all other variables for which data were sought (e.g. participant and intervention characteristics, funding sources). Describe any assumptions made about any missing or unclear information. | Materials and methods; variables included botanical source, quality control, model type, intervention, pharmacokinetics, toxicology, formulation and delivery-related information. |
| Study risk of bias assessment | 11 | Specify the methods used to assess risk of bias in the included studies, including details of the tool(s) used, how many reviewers assessed each study and whether they worked independently, and if applicable, details of automation tools used in the process. | Not performed. Formal risk-of-bias assessment was not conducted because the article is a narrative pharmacological review and no quantitative synthesis was performed. |
| Effect measures | 12 | Specify for each outcome the effect measure(s) (e.g. risk ratio, mean difference) used in the synthesis or presentation of results. | Not applicable. No meta-analysis or pooled effect estimate was performed. |
| Synthesis methods | 13a | Describe the processes used to decide which studies were eligible for each synthesis (e.g. tabulating the study intervention characteristics and comparing against the planned groups for each synthesis (item #5)). | Materials and methods; included studies were grouped narratively according to ASCVD stage, model type, mechanism and translational relevance. Table 1 and Table 2 present study characteristics and translational considerations. |
|  | 13b | Describe any methods required to prepare the data for presentation or synthesis, such as handling of missing summary statistics, or data conversions. | Not applicable. No data conversion or preparation for quantitative synthesis was performed. |
|  | 13c | Describe any methods used to tabulate or visually display results of individual studies and syntheses. | Fig. 1, Table 1, Table 2 and narrative synthesis in Sections 4 and 5. |
|  | 13d | Describe any methods used to synthesize results and provide a rationale for the choice(s). If meta-analysis was performed, describe the model(s), method(s) to identify the presence and extent of statistical heterogeneity, and software package(s) used. | Narrative synthesis only; no meta-analysis was performed. |
|  | 13e | Describe any methods used to explore possible causes of heterogeneity among study results (e.g. subgroup analysis, meta-regression). | Not applicable. No subgroup analysis or meta-regression was performed. |
|  | 13f | Describe any sensitivity analyses conducted to assess robustness of the synthesized results. | Not applicable. No sensitivity analysis was performed. |
| Reporting bias assessment | 14 | Describe any methods used to assess risk of bias due to missing results in a synthesis (arising from reporting biases). | Not performed. Formal reporting-bias assessment was not conducted because no quantitative synthesis was performed. |
| Certainty assessment | 15 | Describe any methods used to assess certainty (or confidence) in the body of evidence for an outcome. | Not performed. Formal certainty-of-evidence assessment was not conducted. |
| **RESULTS** | | |  |
| Study selection | 16a | Describe the results of the search and selection process, from the number of records identified in the search to the number of studies included in the review, ideally using a flow diagram. | Fig. 1; Materials and methods; records identified, duplicate removal, screening and final inclusion are reported. |
|  | 16b | Cite studies that might appear to meet the inclusion criteria, but which were excluded, and explain why they were excluded. | Materials and methods and Fig. 1; records excluded after screening are summarized by eligibility criteria rather than citation-by-citation listing. |
| Study characteristics | 17 | Cite each included study and present its characteristics. | Table 1; cited studies and experimental characteristics are summarized. Additional pharmacokinetic, toxicological and formulation evidence is summarized in Table 2. |
| Risk of bias in studies | 18 | Present assessments of risk of bias for each included study. | Not performed. No formal risk-of-bias table was prepared. |
| Results of individual studies | 19 | For all outcomes, present, for each study: (a) summary statistics for each group (where appropriate) and (b) an effect estimate and its precision (e.g. confidence/credible interval), ideally using structured tables or plots. | Table 1 and Table 2; individual preclinical study models, interventions, readouts and translational implications are presented narratively rather than as effect estimates. |
| Results of syntheses | 20a | For each synthesis, briefly summarise the characteristics and risk of bias among contributing studies. | Sections 4 and 5; narrative synthesis summarizes study characteristics by pathological stage and mechanism. Formal risk-of-bias synthesis was not performed. |
|  | 20b | Present results of all statistical syntheses conducted. If meta-analysis was done, present for each the summary estimate and its precision (e.g. confidence/credible interval) and measures of statistical heterogeneity. If comparing groups, describe the direction of the effect. | Not applicable. No statistical synthesis or meta-analysis was performed. |
|  | 20c | Present results of all investigations of possible causes of heterogeneity among study results. | Not applicable. No investigation of heterogeneity was performed. |
|  | 20d | Present results of all sensitivity analyses conducted to assess the robustness of the synthesized results. | Not applicable. No sensitivity analysis was performed. |
| Reporting biases | 21 | Present assessments of risk of bias due to missing results (arising from reporting biases) for each synthesis assessed. | Not performed. Formal reporting-bias assessment was not conducted. |
| Certainty of evidence | 22 | Present assessments of certainty (or confidence) in the body of evidence for each outcome assessed. | Not performed. Formal certainty-of-evidence assessment was not conducted. |
| **DISCUSSION** | | |  |
| Discussion | 23a | Provide a general interpretation of the results in the context of other evidence. | Sections 4, 5 and Conclusions. |
|  | 23b | Discuss any limitations of the evidence included in the review. | Sections 4 and 5; limitations of plaque-stage evidence, post-ischemic evidence and translational evidence are discussed. |
|  | 23c | Discuss any limitations of the review processes used. | Materials and methods and Conclusions; review-process limitations are addressed through predefined eligibility criteria and narrative synthesis scope. |
|  | 23d | Discuss implications of the results for practice, policy, and future research. | Section 5 and Conclusions; implications for pharmacokinetics, delivery, safety, plaque endpoints and future ASCVD development are discussed. |
| **OTHER INFORMATION** | | |  |
| Registration and protocol | 24a | Provide registration information for the review, including register name and registration number, or state that the review was not registered. | Not registered. This review was not registered in a review registry. |
|  | 24b | Indicate where the review protocol can be accessed, or state that a protocol was not prepared. | No review protocol was prepared. |
|  | 24c | Describe and explain any amendments to information provided at registration or in the protocol. | Not applicable. No protocol amendments were made. |
| Support | 25 | Describe sources of financial or non-financial support for the review, and the role of the funders or sponsors in the review. | Funding statement. |
| Competing interests | 26 | Declare any competing interests of review authors. | Disclosure statement. |
| Availability of data, code and other materials | 27 | Report which of the following are publicly available and where they can be found: template data collection forms; data extracted from included studies; data used for all analyses; analytic code; any other materials used in the review. | Data availability statement. |

Note: This checklist is completed for the submitted Review, which used a structured database search, predefined eligibility criteria and narrative synthesis. No meta-analysis was performed; quantitative synthesis-related items are therefore marked as not applicable or not performed.

*From:*  Page MJ, McKenzie JE, Bossuyt PM, Boutron I, Hoffmann TC, Mulrow CD, et al. The PRISMA 2020 statement: an updated guideline for reporting systematic reviews. BMJ 2021;372:n71. doi: 10.1136/bmj.n71. This work is licensed under CC BY 4.0. To view a copy of this license, visit <https://creativecommons.org/licenses/by/4.0/>
