# Supplementary material for: Protective effects of icariin in atherosclerotic cardiovascular disease: mechanistic basis, post-ischemic protection and translational limitations
Source: Pharm Biol. 2026 Oct 10;64(1):1066–86. doi: 10.1080/13880209.2026.2740872 (PMC13656681; doi:10.1080/13880209.2026.2740872)
Supplement: Supplementary file 3 [file IPHB_A_2740872_SM1048.docx]

**Supplementary Material 2**

**Complete database search strategies**

**Supplementary Table S1. Core ASCVD search strategy for PubMed**

| **No.** | **Search strategy** | **Records retrieved** |
| --- | --- | --- |
| 1 | "icariin"[Supplementary Concept] OR icariin[Title/Abstract] | 1,794 |
| 2 | "Atherosclerosis"[Mesh] OR "Plaque, Atherosclerotic"[Mesh] OR atherosclero*[Title/Abstract] OR arteriosclero*[Title/Abstract] OR atheroma*[Title/Abstract] OR "atherosclerotic plaque"[Title/Abstract] OR "atherosclerotic plaques"[Title/Abstract] OR "atherosclerotic cardiovascular disease"[Title/Abstract] OR ASCVD[Title/Abstract] | 254,260 |
| 3 | "Coronary Artery Disease"[Mesh] OR "Acute Coronary Syndrome"[Mesh] OR "Myocardial Infarction"[Mesh] OR "Myocardial Ischemia"[Mesh] OR "Myocardial Reperfusion Injury"[Mesh] OR "coronary artery disease"[Title/Abstract] OR "coronary heart disease"[Title/Abstract] OR "acute coronary syndrome"[Title/Abstract] OR "ischemic heart disease"[Title/Abstract] OR "ischaemic heart disease"[Title/Abstract] OR "myocardial infarction"[Title/Abstract] OR "myocardial ischemia"[Title/Abstract] OR "myocardial ischaemia"[Title/Abstract] OR "myocardial ischemia-reperfusion"[Title/Abstract] OR "myocardial ischaemia-reperfusion"[Title/Abstract] OR "myocardial reperfusion injury"[Title/Abstract] OR ((post-infarct*[Title/Abstract] OR "post myocardial infarction"[Title/Abstract]) AND remodel*[Title/Abstract]) | 676,910 |
| 4 | "Brain Ischemia"[Mesh] OR "Cerebral Infarction"[Mesh] OR "ischemic stroke"[Title/Abstract] OR "ischaemic stroke"[Title/Abstract] OR "brain ischemia"[Title/Abstract] OR "brain ischaemia"[Title/Abstract] OR "cerebral ischemia"[Title/Abstract] OR "cerebral ischaemia"[Title/Abstract] OR "cerebral infarction"[Title/Abstract] OR "cerebral ischemia-reperfusion"[Title/Abstract] OR "cerebral ischaemia-reperfusion"[Title/Abstract] | 214,218 |
| 5 | "Peripheral Arterial Disease"[Mesh] OR "Carotid Stenosis"[Mesh] OR "peripheral arterial disease"[Title/Abstract] OR "peripheral artery disease"[Title/Abstract] OR "carotid stenosis"[Title/Abstract] OR "carotid atherosclerosis"[Title/Abstract] | 63,021 |
| 6 | "endothelial dysfunction"[Title/Abstract] OR "vascular calcification"[Title/Abstract] OR "foam cell"[Title/Abstract] OR "foam cells"[Title/Abstract] OR "oxidized low-density lipoprotein"[Title/Abstract] OR "oxidised low-density lipoprotein"[Title/Abstract] OR ox-LDL[Title/Abstract] OR oxLDL[Title/Abstract] | 62,821 |
| 7 | #2 OR #3 OR #4 OR #5 OR #6 | 1,112,570 |
| 8 | #1 AND #7 | 89 |

**Supplementary Table S2. Core ASCVD search strategy for Web of Science Core Collection**

| **No.** | **Search strategy** | **Records retrieved** |
| --- | --- | --- |
| 1 | TS=(icariin) | 2,098 |
| 2 | TS=(atherosclero* OR arteriosclero* OR atheroma* OR "atherosclerotic plaque*" OR "atherosclerotic cardiovascular disease*" OR ASCVD) | 219,652 |
| 3 | TS=("coronary artery disease" OR "coronary heart disease" OR "acute coronary syndrome" OR "ischemic heart disease" OR "ischaemic heart disease" OR "myocardial infarction" OR "myocardial ischemia" OR "myocardial ischaemia" OR "myocardial ischemia-reperfusion" OR "myocardial ischaemia-reperfusion" OR "myocardial reperfusion injury" OR ((post-infarct* OR "post myocardial infarction") AND remodel*)) | 449,456 |
| 4 | TS=("ischemic stroke" OR "ischaemic stroke" OR "brain ischemia" OR "brain ischaemia" OR "cerebral ischemia" OR "cerebral ischaemia" OR "cerebral infarction" OR "cerebral ischemia-reperfusion" OR "cerebral ischaemia-reperfusion") | 166,178 |
| 5 | TS=("peripheral arterial disease" OR "peripheral artery disease" OR "carotid stenosis" OR "carotid atherosclerosis") | 40,358 |
| 6 | TS=("endothelial dysfunction" OR "vascular calcification" OR "foam cell*" OR "oxidized low-density lipoprotein" OR "oxidised low-density lipoprotein" OR ox-LDL OR oxLDL) | 74,545 |
| 7 | #2 OR #3 OR #4 OR #5 OR #6 | 810,204 |
| 8 | #1 AND #7 | 115 |

**Supplementary Table S3. Targeted translational search strategy for PubMed**

| **No.** | **Search strategy** | **Records retrieved** |
| --- | --- | --- |
| 1 | "icariin"[Supplementary Concept] OR icariin[Title/Abstract] | 1,794 |
| 2 | pharmacokinetic*[Title/Abstract] OR bioavailability[Title/Abstract] OR absorption[Title/Abstract] OR distribution[Title/Abstract] OR metabolism[Title/Abstract] OR metabolic[Title/Abstract] OR biotransformation[Title/Abstract] OR excretion[Title/Abstract] OR disposition[Title/Abstract] OR toxicolog*[Title/Abstract] OR toxicity[Title/Abstract] OR safety[Title/Abstract] OR "adverse effect"[Title/Abstract] OR "adverse effects"[Title/Abstract] OR formulation*[Title/Abstract] OR "drug delivery"[Title/Abstract] OR solubility[Title/Abstract] OR release[Title/Abstract] OR nanoparticle*[Title/Abstract] OR nanocarrier*[Title/Abstract] | 5,944,264 |
| 3 | #1 AND #2 | 581 |

**Supplementary Table S4. Targeted translational search strategy for Web of Science Core Collection**

| **No.** | **Search strategy** | **Records retrieved** |
| --- | --- | --- |
| 1 | TS=(icariin) | 2,098 |
| 2 | TS=(pharmacokinetic* OR bioavailability OR absorption OR distribution OR metabolism OR metabolic OR biotransformation OR excretion OR disposition OR toxicolog* OR toxicity OR safety OR "adverse effect*" OR formulation* OR "drug delivery" OR solubility OR release OR nanoparticle* OR nanocarrier*) | 9,006,536 |
| 3 | #1 AND #2 | 800 |

**Supplementary Table S5. Targeted botanical source and quality-control search strategy for PubMed**

| **No.** | **Search strategy** | **Records retrieved** |
| --- | --- | --- |
| 1 | "icariin"[Supplementary Concept] OR icariin[Title/Abstract] OR Epimedium[Title/Abstract] OR "Epimedii Folium"[Title/Abstract] | 2,693 |
| 2 | botanical[Title/Abstract] OR taxonomy[Title/Abstract] OR species[Title/Abstract] OR source[Title/Abstract] OR "plant material"[Title/Abstract] OR "quality control"[Title/Abstract] OR quality[Title/Abstract] OR standardization[Title/Abstract] OR standardisation[Title/Abstract] OR fingerprint*[Title/Abstract] OR extraction[Title/Abstract] OR purification[Title/Abstract] OR pharmacopoeia[Title/Abstract] OR pharmacopeia[Title/Abstract] OR "flavonoid content"[Title/Abstract] OR "chemical profile"[Title/Abstract] | 4,521,887 |
| 3 | #1 AND #2 | 638 |

**Supplementary Table S6. Targeted botanical source and quality-control search strategy for Web of Science Core Collection**

| **No.** | **Search strategy** | **Records retrieved** |
| --- | --- | --- |
| 1 | TS=(icariin OR Epimedium OR "Epimedii Folium") | 2,964 |
| 2 | TS=(botanical OR taxonomy OR species OR source OR "plant material" OR "quality control" OR quality OR standardization OR standardisation OR fingerprint* OR extraction OR purification OR pharmacopoeia OR pharmacopeia OR "flavonoid content" OR "chemical profile") | 7,779,873 |
| 3 | #1 AND #2 | 800 |
